# Two-photon axotomy and time-lapse confocal imaging in live zebrafish embryos

**DOI:** 10.3791/1129

**Published:** 2009-02-16

**Authors:** Georgeann S. O'Brien, Sandra Rieger, Seanna M. Martin, Ann M. Cavanaugh, Carlos Portera-Cailliau, Alvaro Sagasti

**Affiliations:** Department of Molecular Cell and Developmental Biology, University of California, Los Angeles; Departments of Neurology and Neurobiology, University of California, Los Angeles

## Abstract

Zebrafish have long been utilized to study the cellular and molecular mechanisms of development by time-lapse imaging of the living transparent embryo.  Here we describe a method to mount zebrafish embryos for long-term imaging and demonstrate how to automate the capture of time-lapse images using a confocal microscope.  We also describe a method to create controlled, precise damage to individual branches of peripheral sensory axons in zebrafish using the focused power of a femtosecond laser mounted on a two-photon microscope.  The parameters for successful two-photon axotomy must be optimized for each microscope.  We will demonstrate two-photon axotomy on both a custom built two-photon microscope and a Zeiss 510 confocal/two-photon to provide two examples.

Zebrafish trigeminal sensory neurons can be visualized in a transgenic line expressing GFP driven by a sensory neuron specific promoter ^1^.  We have adapted this zebrafish trigeminal model to directly observe sensory axon regeneration in living zebrafish embryos.  Embryos are anesthetized with tricaine and positioned within a drop of agarose as it solidifies.  Immobilized embryos are sealed within an imaging chamber filled with phenylthiourea (PTU) Ringers.  We have found that embryos can be continuously imaged in these chambers for 12-48 hours.  A single confocal image is then captured to determine the desired site of axotomy.  The region of interest is located on the two-photon microscope by imaging the sensory axons under low, non-damaging power.  After zooming in on the desired site of axotomy, the power is increased and a single scan of that defined region is sufficient to sever the axon.  Multiple location time-lapse imaging is then set up on a confocal microscope to directly observe axonal recovery from injury.

**Figure:**
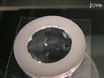


## Protocol

### Part 1: Mounting zebrafish embryos for long-term imaging

1. Prepare 1% low melt agarose solution for embedding.  Dissolve agarose in DI water by heating in a microwave, aliquot into small tubes and store in a heating block at 42 degrees Celsius.
2. Select embryos for imaging and remove their chorions by gently pulling the chorion apart with forceps.  Embryos can be placed into a 5% PTU Ringers solution at 22-24 hours post fertilization (hpf) to inhibit the formation of pigment.  This improves the clarity of imaging and is essential for performing two-photon axotomies of sensory axons.  If the natural pigment is allowed to form, autofluorescence obscures axons on the two-photon microscope. Ringers solution for zebrafish contains 116 mM NaCl, 2.9 mM KCl, 1.8 mM CaCl_2_, and 5mM HEPES, pH 7.2.
3. Anesthetize embryos by adding ~0.02% tricaine to the PTU Ringers.  Make sure animals are not responsive to touch before proceeding.
4. Prepare the long-term imaging chamber by applying vacuum grease to one side of a glass or Teflon ring and fixing it onto a glass cover slip.
5. Transfer one embryo into the 42 degree agarose solution with a glass pipet, taking care not to transfer much of the PTU Ringers, then transfer the embryo with one drop of agarose onto a prepared cover slip.
6. Position the embryo as desired as the agarose hardens.  Keep in mind that the imaging chamber will be flipped when you are done so that the cover slip will be the top surface.
7. If you have a motorized stage and can image multiple locations at once, repeat steps 1.4-1.6 for each embryo.
8. Allow the agarose to completely solidify, then fill the ring with 0.02% tricaine PTU Ringers.
9. Apply vacuum grease to the other side of the ring(s), then cover the ring(s) with a glass slide. Flip the sealed chamber(s) over.  These chambers can be used for imaging on upright or inverted microscopes.

### Part 2: Two-photon axotomy using a custom built two-photon microscope with a Chameleon Ti-Sapphire laser

1. Prepare for imaging. Place the mounted embryo(s) on a slide holder under the microscope.  Focus on one embryo with a 40X (0.8 NA) water objective.  Turn on the laser.  We have been able to reproducibly visualize and damage GFP expressing neurons using the following parameters. To visualize axons at a non-damaging power, set laser to a wavelength of 910 nanometers (nm) and a power of 30 milliwatts (mW listed are the amount of power at the sample).  Open the imaging software.  We use ScanImage Software developed in Karel Svoboda's laboratory ^2, 3^.
2. Press focus to scan the embryo with the two-photon laser, locate the axon you wish to axotomize, and capture an image of this axon.  Mark the first and last Z positions, acquire the image, and make a maximum projection of the Z stacks.
3. Zoom in 70X on the branch of the axon you wish to axotomize and stop scanning.  Increase the mW at the sample to the damaging power of 180 mW, and perform a single scan with the laser. We do this by setting the number of Z slices to 1 and pressing “Grab”.  This should be sufficient to sever the axon.  See discussion for methods to optimize this procedure for your microscope and experimental goal.
4. Zoom out, reduce the power to 30 mW and take an image.

### Part 3: Two-photon axotomy on Zeiss 510 confocal/two-photon microscope

1. Place mounted embryo onto the stage and bring it into focus using a 25X water objective or other suitable objective.
2. Turn on the two-photon (910 nm) and Argon (488 nm) lasers in a multi-track setting so that it is possible to switch from one to the other. Although both lasers are used to detect GFP, the two-photon emission is visualized with red, and the argon laser emission with green to differentiate the two.
3. Use the Argon laser to identify an axon to injure. Under Z settings mark the first and last optical sections. Take a confocal image and create a maximum projection of the Z stack.
4. Choose the region of the axon to be injured and bring this region into focus. Turn off the Argon laser and turn on the two-photon laser.  Scan the specimen with the two-photon at a laser intensity of ~9% transmission to make sure that the axon is still in focus.
5. Click the “stop” button so that the crop tool will be available. Use crop to zoom in on the area of interest. Usually we zoom to ~70X (zoom can be checked under the “mode” tab). Under the “channels” tab change the intensity of the two-photon from ~9% transmission to 15-20% transmission.
6. To sever an axon activate the “fast XY” button for about 1 second and then press the stop button to avoid excess damage. The axon should be seen as scattered debris if the procedure worked.
7. To ensure that the axon was damaged switch back to the 488 nm Argon laser, take another confocal image, and create a maximum projection.

### Part 4: Confocal time-lapse imaging on Zeiss LSM 510

1. Prepare for imaging. If you are using a heated stage, be sure to turn it on at least 30 minutes before setting up your time-lapse movie.  Place the mounted embryo(s) on a slide holder under the microscope.  Focus on one embryo with desired air objective.  We use a 20X, 0.5 NA objective.  Open Zeiss LSM 510 imaging software.  Turn on the appropriate lasers and set up the desired configuration. We will now describe a detailed protocol for long term imaging with Zeiss LSM Multi Time software.  These methods can be adapted for use on your own microscope with your imaging software.
2. Define the position and configuration of your first embryo in the Multi Time window. Open the scan, stage, and Multi Time windows.  Activate fast scan on the scan window.  Move the stage to the desired XY position.  Mark the first and last Z positions in the scan window.  Press stop, then Mid, also in the scan window.  Wait for the scan of the middle Z slice to finish, then press mark position in the stage window.  If you are only imaging one embryo, click on the “Single Location” tab in the Multi Time window.  Click on “Replace XYZ” and then on “Save Configuration”.  Agree when asked to overwrite the previous configuration. Proceed to step 4.4. If you have a mechanized stage, you can set up multiple locations to image multiple embryos.  In this case, you should select the “Multiple Locations” tab before defining the XYZ and configuration for your first location.  Also, be sure that the pull-down menu just below the “Multiple Locations” tab is on location 1, and that the pull-down menu in the configuration section says multi loc 1, before you click on save configuration.
3. Define the position and configuration of your remaining embryos in the Multi Time window.  Locate your next embryo, then press fast scan, move the stage into the desired XY position, and mark your first and last Z positions in the scan window.  Press stop, then Mid, also in the scan window.  Wait for the scan of the middle Z slice to finish, then press mark position in the stage window.  Click on “Replace XYZ”.   Check that the pull-down menu just below the “Multiple Locations” tab is on location 2, and that the pull-down menu in the configuration section says multi loc 2, then click on “Save Configuration”.  Agree when asked to overwrite the previous configuration.  Repeat this process for the remaining embryos.
4. Set up the parameters for image acquisition in the Multi Loc window.  Choose the GR-G-L option in the “List of Blocks” section, then enter the desired number of group repetitions.  This is the number of times the confocal will acquire an image at each location.  Enter the desired wait interval in the Parameters section.  This is the amount of time from the start of one imaging repetition to the start of the next repetition.  Select “ZStackXY” in the configuration section.  Enter you base file name in the bottom section.  Click on “Select Image DB” and choose your mdb.  Click on the “Options” button.  Select your mdb folder to save temp files.  Check “keep final image open”, “save final image”, and “middle of the z stack”. Select wait interval.  Click ok to close the options window.  Click on “start time”.  Leave Multi Time window open for duration of imaging.
5. Analyze your data.  When the time-lapse imaging has finished, the Multi Time software will compile all of your temporary files into a summary file for each location.  If you want to stop the movie before it is complete, be sure to press “Finish” instead of “Stop”, so that a summary file will be created.  The temp files and summary files should be automatically saved to the designated mdb folder.  From the LSM software menu, click on “3D View”, then “Projection”.  With your summary file selected in the pull-down menu, click on “Apply”.  This will generate maximum projections of the Z stack for each confocal image.  On the LSM software menu, click on “File”, then “Export”.  Save the maximum projections as a series of tif files.  You may then create a movie out of the series of tif files using ImageJ or QuickTime Pro.  Axons in the LSM images can be traced in three dimensions using image analysis software (e.g. NeuroLucida from MicroBrightfield) to generate detailed quantitative information about axon morphology.

### Part 5: Representative results

A successful experiment will result in an accurate representation of the cellular dynamics of axon recovery from injury.  Your embryos will be healthy after imaging, with no visible degeneration and a strong heartbeat.  The axotomy should result in precise damage, only severing the defined branch of the axon.  There should be no injury to surrounding axons and minimal cell death.  We believe we see the death of a single epidermal cell directly over the site of axotomy in ~50% of experiments.  If you observe more damage, you should optimize your two-photon protocol as described in the discussion.

## Discussion

We have used the methods described to precisely axotomize peripheral sensory axons and to directly observe regeneration in the living zebrafish embryo.  Long-term time-lapse confocal imaging in zebrafish can be used to observe many developmental processes *in vivo*.  The two-photon axotomy procedure described can be modified for many different experimental goals.  We have used the same general procedure to ablate entire trigeminal sensory neuron cell bodies, by zooming in on the cell body rather than on a branch of the peripheral axon.  Any cell type identifiable with fluorescence can be precisely damaged or ablated with the focused power of the femtosecond laser.  We were inspired to perfect these techniques for the zebrafish system by previous studies in several other systems where pulsed lasers were used to create localized damage or to ablate cells^4,5,6^.  In control experiments we confirmed that axotomy is extremely precise:  we have never damaged nearby axons, even when they are branches of the same cell, and only occasionally damaged epithelial cells in close juxtaposition to axons.  This specificity can be explained by the fact that intensity from two-photon laser excitation drops off quadratically with distance from the focal point^3,7^.  Moreover, since energy emitted from the excited fluorophore contributes to photodamage, surrounding unlabeled cells are likely to be spared.

The laser power required to damage an axon may vary depending on the set up of the laser, the depth of the tissue, and your experimental goal. If you wish to damage axons deeper in the embryo, more power will be required.  It is best to attempt the axotomy at a low power, and then incrementally increase the power until you find the amount that will sever the axon.  Once you have determined the appropriate amount of laser power for axotomy, you should be able to reproducibly use this laser power to cause local tissue damage.  If you notice that over time more power is required to create an axotomy, the laser and microscope may require maintenance (for example, the laser may be out of alignment).  Make sure your laser is properly aligned, clean your objective, and check the mirrors.
